# Editorial

**Published:** 1899-01

**Authors:** 


### HOMŒOPATHIC PHYSICIAN,

A MONTHLY JOURNAL OF

HOMŒOPATHIC MATERIA MEDICA AND CLINICAL MEDICINE.

94

If our school ever gives up the strict inductive method of Hahnemann, we are lost, and deserve only to be mentioned as a caricature in the history of medicine."—CONSTANTINE HERING.

Vol. XIX. JANUARY, 1899. No. 1.

### EDITORIAL.

PULSATILLA.—We give herewith the remaining keynotes of Pulsatilla as formulated by Dr. Henry N. Guernsey, thus closing up the subject of Pulsatilla.

Retention of urine, with redness, heat, and soreness of the vesical region externally. Continual pressure on the bladder without desire to urinate. Desire to urinate with drawing in the abdomen. Involuntary urination when sitting or walking. After urinating, spasmodic pain in neck of bladder, extending to pelvis and thighs. Frequent and almost ineffectual urging to urinate, with cutting pain. Sensation as if the ears were stopped up. Sensation as if the eyes were covered with a mist, or as if the dimness would be removed by rubbing something off the eyes.

Better from cold things and worse from warm. Much weeping even at answering a question.

Discharge in threatened abortion is arrested for a while, then returns with redoubled violence. This cessation and renewal is frequently repeated. Labor pains worse toward evening. Milk suddenly disappears from the breasts.

The scanty lochial discharge remaining is milky. Fever without thirst.

The breasts are much swollen and rheumatic pains extend to the muscles of the chest, shoulders, neck, and axillæ, and down the arms. The uterus seems inactive. The labor pains excite palpitations, suffocations, and fainting spells, unless the doors and windows are wide open.

Alternating hemorrhage. She weeps at every nursing. The pain from nursing extends into the chest, up into the neck and down the back. In puerperal convulsions the countenance is cold, clammy, and pale. Loss of consciousness and of motion. Sterterous breathing and full pulse.

The labor pains are deficient, irregular, and sluggish.

Leucorrhœa, worse at night. No two stools alike, they are so changeable.

For a time the child seems better and then suddenly gets worse. The child always seems better in the open air.

